# Development of a *gp60*-subtyping method for *Cryptosporidium felis*

**DOI:** 10.1186/s13071-020-3906-9

**Published:** 2020-01-23

**Authors:** Laura Rojas-Lopez, Kristin Elwin, Rachel M. Chalmers, Heidi L. Enemark, Jessica Beser, Karin Troell

**Affiliations:** 1 Public Health Agency of Sweden, 171 82 Solna, Sweden; 2 Department of Cell and Molecular Biology, Uppsala University, Box 596, 751 24 Uppsala, Sweden; 3 Cryptosporidium Reference Unit, Public Health Wales Microbiology and Health Protection, Singleton Hospital Sgeti, Swansea, SA2 8QA UK; 4 Swansea University Medical School, Swansea University, Grove Building, Singleton Park, Swansea, SA2 8PP UK; 5 National Veterinary Institute, Ullevålsveien 68, 0454 Oslo, Norway; 6 National Veterinary Institute, 751 89 Uppsala, Sweden

**Keywords:** Cryptosporidiosis, Molecular typing, Zoonotic transmission, 60-kDa glycoprotein, Source tracking, Genetic variability, Epidemiological marker

## Abstract

**Background:**

Feline cryptosporidiosis is an increasing problem, especially in catteries. In humans, close contact with cats could be a potential source of infection although the risk of contracting cryptosporidiosis caused by *Cryptosporidium felis* is considered to be relatively low. Sequencing of the 60-kDa glycoprotein gene is a commonly used tool for investigation of the genetic diversity and transmission dynamics of *Cryptosporidium* species. However, until now the sequence of *gp60* from *C. felis* has not been available and genotyping has been limited to less discriminatory markers, such as *18S* rRNA, COWP and HSP70.

**Methods:**

We have identified the *gp60* orthologue within the genome sequence of *C. felis*, and used the sequence to design a nested PCR for subtyping purposes. A total of 128 clinical isolates of both feline and human origin, were used to evaluate the marker.

**Results:**

Sequence analysis revealed large variations between the different samples. The *C. felis gp60* lack the characteristic serine-tract found in many other cryptosporidian orthologues, instead it has an insertion of variable length (361–742 nt). Also, two cases of suspected zoonotic transmission of *C. felis* between cats and humans were successfully confirmed.

**Conclusions:**

We have identified the *gp60* gene in *C. felis* and show how this highly variable marker can be used in epidemiological investigations.
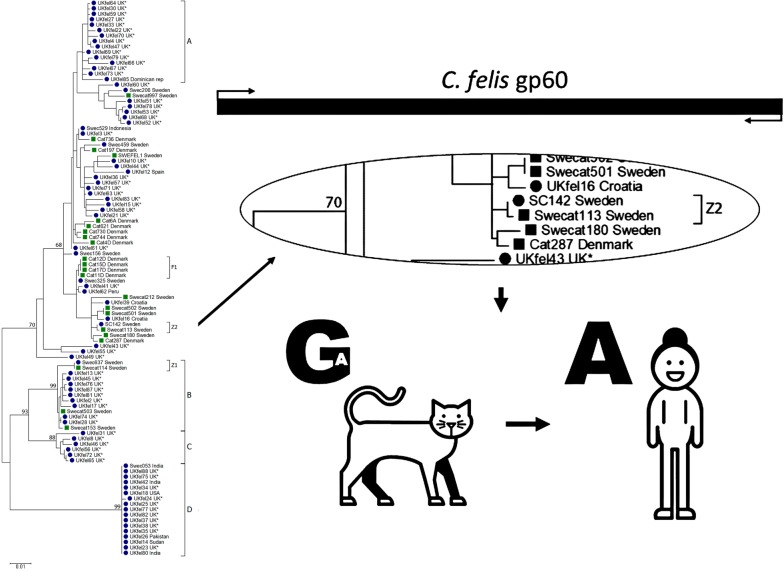

## Background

*Cryptosporidium* species are significant pathogens that infect a wide range of vertebrate hosts, causing a considerable burden of gastrointestinal disease [1]. Human cryptosporidiosis is mainly caused by *Cryptosporidium hominis* and *Cryptosporidium parvum*, the latter also being an important zoonotic pathogen [2]. However, the use of molecular techniques has shown that other species of *Cryptosporidium* also have zoonotic potential, including, but not limited to, *Cryptosporidium meleagridis*, *Cryptosporidium cuniculus*, *Cryptosporidium ubiquitum*, *Cryptosporidium canis* and *Cryptosporidium felis* [2].

The close association between owners and their companion animals has thus a potentially significant role in the zoonotic transmission of parasites [3, 4]. Even though the population-based risk of contracting *Cryptosporidium* spp. from domestic cats is considered to be relatively low [5, 6], there have been several reports showing human infection with *C. felis* [2, 7–12]. In 2015, one investigation of a possible zoonotic transmission between a cat and its owner showed that, although the *C. felis* isolated from both the cat and the owner had identical sequences at several markers, so did all other *C. felis* samples from unrelated cats [11]. This highlighted the need for a higher-resolution subtyping method for this *Cryptosporidium* species.

The 60-kDa glycoprotein gene (*gp60*) is the most commonly used marker for subtyping of *Cryptosporidium* spp., mainly *C. parvum* and *C. hominis*, and has been a useful tool to study sources of infection, genetic diversity and host adaptation. Recently, subtyping methods targeting the *gp60* gene of *C. meleagridis*, *C. ubiquitum*, *C. viatorum*, *Cryptosporidium* skunk genotype and chipmunk genotype I have been developed [13–17]. These additional subtyping tools are needed since the PCR primers designed on *C. parvum* and *C. hominis* sequences are not capable of amplifying efficiently the *gp60* gene of these divergent *Cryptosporidium* spp. [18].

Given that *C. felis* is relatively genetically distant from *C. hominis* and *C. parvum*, the *gp60* orthologue has previously not been identified. In this study, the *gp60* gene of *C. felis* was identified by massive parallel sequencing of a clinical *C. felis* sample and PCR primers were developed and used to characterize *C. felis* isolates retrieved from both humans and cats.

## Methods

### Samples

DNA extracts from 128 *C. felis*-positive fecal samples were included in the present study; 93 samples were from humans and 35 from cats (Table 1), and spanned the period 2000 to 2015. Human samples used for screening were collected either by the Public Health Agency of Sweden (eight samples) or as part of national molecular epidemiology in the Cryptosporidium Reference Unit in the UK [19] (85 samples) (Table 1). All the samples were originally identified as containing *C. felis* by restriction fragment length polymorphism (RFLP) analysis and/or by sequencing of the partial *18S* rRNA gene [20]. Samples related to foreign travel were identified either from the submission form or from a routinely administered patient questionnaire (UK) [12], or from information obtained through the national mandatory notifications system (SmiNet) or the local department of Communicable Disease Control (Sweden). Samples from patients travelling to Croatia, Dominican Republic, India, Indonesia, Pakistan, Peru, Spain, Sudan and USA were included in addition to samples from those that had not travelled (Table 1).

**Table 1:** Origin and history of *Cryptosporidium felis* DNA extracts investigated in the study

Host	Collection	Origin	*n*	Travel history
Cat	Sweden	Domestic	11	
Cat	Denmark	Domestic and feral	24	
Human	Sweden	Domestic	6	
Human	Sweden	Travel-related	2	India, Indonesia
Human	UK	Domestic or travel history unknown	73	
Human	UK	Travel-related	12	Croatia, Dominican Republic, USA India, Pakistan, Peru, Spain, Sudan,

*Abbreviation*: n, number of samples

Feline samples were obtained from the National Veterinary Institute (NVI) and the Public Health Agency in Sweden and from the Technical University of Denmark, National Veterinary Institute (DTU-VET) in Denmark. At NVI the samples were collected from routine diagnosis of gastroenteritis while the samples at the Public Health Agency were collected as part of a study or because zoonotic transmission was suspected. The Danish samples originated from domestic and feral cats from veterinary clinics, cat shelters and breeders in the Copenhagen Metropolitan Region. The samples were collected as part of an epidemiological study of cryptosporidiosis and giardiasis in cats. A total of 24 samples were included from Denmark and 11 from Sweden (Table 1). All of these samples had previously been tested positive for *Cryptosporidium* by immunofluorescence microscopy and *C. felis* had been detected in some of the samples by PCR and sequencing of the *18S* and/or HSP70 loci (Enemark et al. unpublished). All samples included in the present study were confirmed positive for *C. felis* by PCR and sequencing of *18S* rRNA locus [20].

### Subtyping marker

Initially, a draft genome sequence of *C. felis*, obtained from a clinical isolate from a Swedish household cat with diarrhea (unpublished data), was screened for *gp60* using tBLASTx homology searches, with *Cryptosporidium* orthologues as seed sequences. The *C. felis* draft genome sequence comprised 8.74 Mbp in 109 contigs. However, this approach generated very weak hits. Instead we aligned amino acid sequences of *gp60* orthologues using Clustal X, and the resulting alignment was searched to identify the most conserved motif. A homology search using amino acid sequence FVMWFGEGTPVATLKCGGY identified a homologous motif, FTVWFDGGIPITTIGCG (e = 0.57), embedded in a predicted coding sequence of 1899 nt, 633 aa. Using the complete 1899 nt to perform a homology search of sequences from various species of *Cryptosporidium* yielded a best hit to an uncharacterized *Cryptosporidium* sequence (GenBank: AJW72319) (47% identity E = 6e^−53^) followed by a *gp60* ortholog in *C. viatorum* (GenBank: AQY61281, 46% identity E = 3e^−51^). The *C. felis gp60* candidate sequence was aligned to a set of orthologues using Clustal X and the most conserved part of the alignment were used to design primers for nested PCR.

### Primer design

A conserved region of the *gp60* gene was selected to design primer sets for nested PCR. PCR amplification was performed using the primers GP60CF_F1 (5′-TTT CCG TTA TTG TTG CAG TTG CA-3′) and GP60CF_R1 (5′-ATC GGA ATC CCA CCA TCG AAC-3′) for primary reactions, and GP60CF_F2 (5′-GGG CGT TCT GAA GGA TGT AA-3′) and GP60CF_R2 (5′-CGG TGG TCT CCT CAG TCT TC-3′) for secondary reactions with PCR products expected ~ 1200 bp and 900 bp respectively based on the genome sequence of *C. felis* (unpublished data).

### PCR screening

Nested PCR was carried out in a total volume of 20 μl of PCR mixture, containing Maxima Hot Start 2× PCR master mix (Thermo Fisher Scientific, Waltham, USA), 0.5 μM of each primer, and 1 to 3 μl of extracted DNA. For the primary PCR 0.4 μl of bovine serum albumin (20 mg/ml) (Thermo Fisher Scientific) was added to the mix. For the secondary PCR, 1 μl of the primary PCR product was used. Reaction conditions were initial denaturation of 95 °C for 4 min followed by 35 cycles of 95 °C for 30 s, 55 °C for 30 s and 72 °C for 1.5 min, followed by a final extension step at 72 °C for 7 min.

### DNA sequence analysis

Positive products of the secondary *gp60* PCR were purified using ExoSAP-IT (Thermo Fisher Scientific) and Sanger sequenced bidirectionally (BigDye chemistry; Applied Biosystems, Waltham, USA) using the primers designed for secondary PCR. Sequences obtained were manually edited and analyzed using BioEdit sequence alignment editor (version 7.0.9.0). Subsequently, nucleotide sequences were aligned and compared with the full *C. felis gp60* gene sequences using CLUSTALW algorithm [21].

Nucleotide as well as amino acid sequences were aligned using T-coffee alignment software [22] and short sequences were removed to not affect the result of the tree. The final analysis involved 102 nucleotide sequences. Ends of all sequences were trimmed, resulting in an alignment covering 1291 bases. A phylogenetic model selector as implemented in MEGA 6 [23] was used to test which model best fitted the data. A phylogenetic tree was inferred in MEGA 6 by using the Maximum Likelihood method based on the Tamura 3-parameter model [24]. A discrete Gamma distribution was used to model evolutionary rate differences among sites (1 category (+G, parameter = 0.1703) [24]). The rate variation model allowed for some sites to be evolutionarily invariable (+I, 75.8281% sites). The robustness of the phylogeny was tested with 1000 bootstrap replicates. In order to avoid over-interpretation, only confidence levels for well-supported nodes are shown.

To illustrate the difference between *Cryptosporidium* species within the *gp60*, an amino acid alignment using BioEdit sequence alignment editor (7.0.9.0) was made comparing reference sequences of *C. hominis*, *C. parvum*, *C. viatorum*, *C. meleagridis*, *C. fayeri*, *C. ubiquitum*, *Cryptosporidium* chipmunk genotype I and *C. felis.* All the sequences were downloaded from the NCBI GenBank database with the exception of *C. felis* in which case the full *gp60* sequences (SWEFEL1) generated by massive parallel sequencing were used.

### Nucleotide sequence accession numbers

Nucleotide sequences of *C. felis* subtype variants identified have been deposited in the GenBank database under the accession numbers MH240831-MH240912 (Additional file 1: Table S1).

## Results

### *Cryptosporidium felis gp60* gene and sample characteristics

**Fig. 1:**
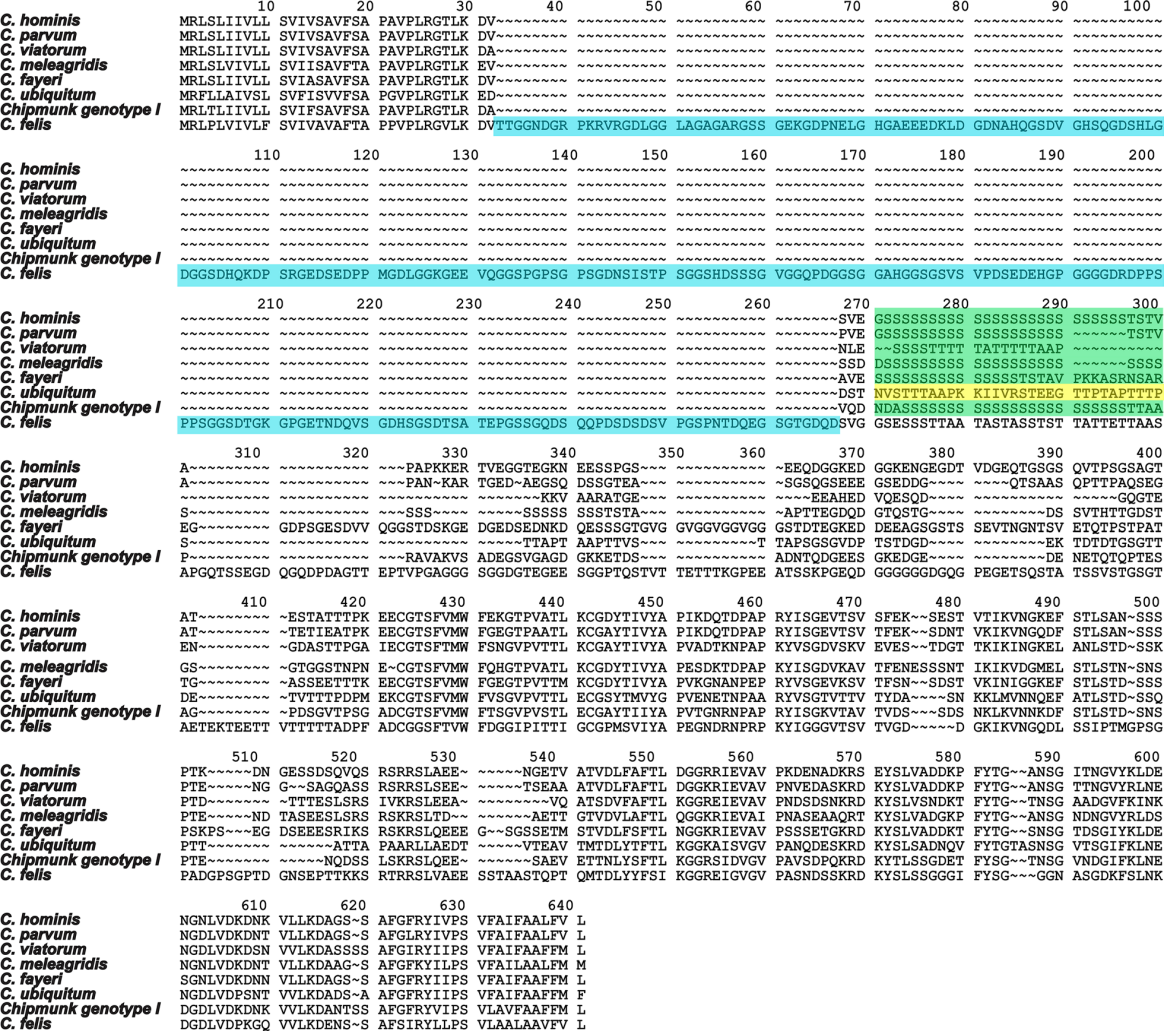
Features of the *gp60* gene of *C. felis*. Amino acid alignment illustrating the differences in the *gp60* between *Cryptosporidium* species. The insertion present in *C. felis* is highlighted in blue and the serine repeats region characteristic of other *Cryptosporidium* species is highlighted in green. *Cryptosporidium ubiquitum* lacks the typical S repeat, the region is highlighted in yellow

A comparison of the region harbouring the *C. felis gp60* orthologue revealed synteny to *C. parvum* and *C. hominis*, further strengthening the hypothesis that a true *gp60* orthologue had been identified. To characterize the *C. felis gp60* gene, 128 *C. felis* positive DNA samples from different locations and origins were analyzed (Table 1). A total of 102 samples were successfully amplified and sequenced (80 human samples and 22 cat samples) whereof 82 unique sequences were obtained. Negative amplification attempts were most likely due to extensive DNA degradation in old fecal samples or those not stored at optimal conditions. Samples positive by nested *gp60* PCR showed fragment lengths ranging from 896 bp to 1285 bp, as predicted, but differences in length could not be linked to host or origin of samples. All *C. felis* sequences lacked the characteristic serine-tract of many *gp60* orthologues identified in cryptosporidia, instead an insertion (Fig. 1, highlighted in blue), of variable length (361–742 nt), with no homology to the serine tracts was present. As previously reported in the *gp60* of *C. viatorum* [16] some randomly occurring double peaks were observed in 10/102 (10%) samples.

In a general overview, more *C. felis* samples from humans were from men (*n* = 44) than women (*n* = 36). The age of the patients ranged from 1 to 83 years-old (median: 26 years-old; mean: 27 years-old). When it comes to the cats the age ranged from below 0.5 to 10 years-old and the majority were domestic cats. A few were stray or feral cats of which the age was unknown. Four samples from cats representing a feline outbreak in a breeding establishment (F1) were included (Enemark et al. unpublished), and so were samples from both cat and owner in two zoonotic cases (Z1 and Z2). (Additional file 1: Table S1)

### Phylogeny

**Fig. 2:**
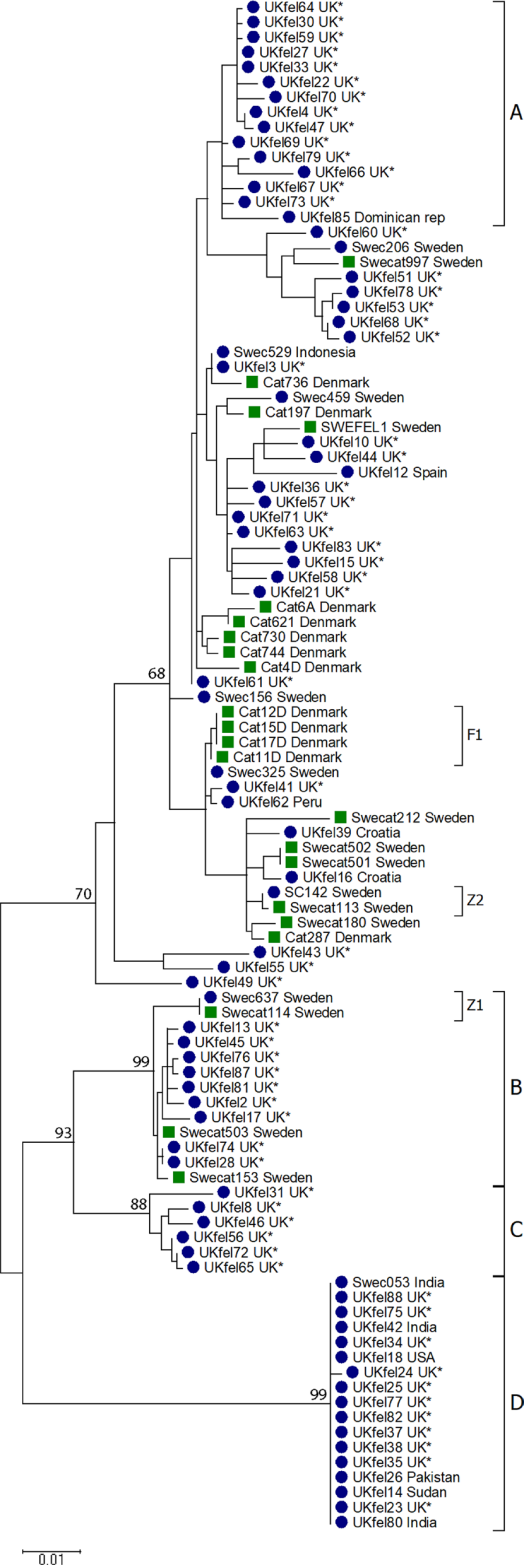
Phylogenetic analysis of 102 *gp60* sequences from *C. felis* isolated from humans and cats. Blue dots indicate sample from human and green squares indicate sample from cat. All cases with known travel history have been indicated while samples with unknown travel history are marked with an asterisk

Nucleotide and amino acid sequences were used to calculate phylogenies using the Maximum Likelihood method based on the Tamura 3-parameter model. However, the data did not yield any fully-resolved, well-supported trees. Three well supported (bootstrap ≥ 80) clusters were identified, B–D (Fig. 2). Two of the supported clusters (C and D) and one additional cluster (A, Fig. 2) consisted of sequences isolated from human cases of cryptosporidiosis only, and one cluster (B) was mixed, comprising sequences from a suspected zoonotic transmission between a cat and its’ owner. Cluster A comprised 15 sequences from human cases, 14 from the UK and one travel-related, from the Dominican Republic; the sex distribution in this cluster was slightly dominated by female patients (*n* = 9). Cluster B comprised 13 sequences isolated from both cats and humans, with the majority being male (7/10). Cluster C contained six sequences, all isolated from UK patients. These sequences represent four male and two females.

Cluster D comprised 17 sequences from human hosts. More cases were male (*n* = 16) than female (*n* = 1) [23] and stemmed mainly from the UK. However, there were also several cases that had travelled to India or Pakistan in Cluster D. The sequences within Cluster D had almost no variation (0–3 nt) and this was the most distinct group in our dataset.

### Connected cases

All four cat sequences from the outbreak in a Danish cattery grouped together in the phylogenetic tree (Fig. 2, F1). Three of the isolates, from an adult cat and two kittens, were identical, whereas an isolate from a third kitten differed by 37 nucleotides (of which 36 nucleotides represents indels and one a SNP).

**Fig. 3:**
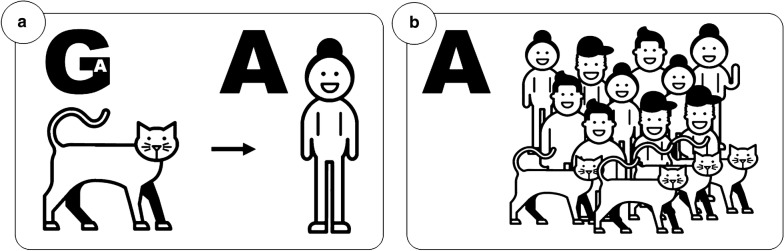
**a** In zoonotic case 2 (Z2) one nucleotide in the *gp60* sequence clearly showed how a dominant variant of *C. felis* shifted to another when transmitted from one host (cat) to another (human). The nucleotide in position 833 differed between the cat (G) and the owner (A). Massive parallel sequencing of the cat sample, covering the named position 62 times, showed that one of the 62 reads indeed had the adenine (A) found in the human sample. **b** All other cats and humans in the study had A in this specific position

Within our dataset, two zoonotic transmissions between cat and owner were further confirmed; in both cases the owner and cat grouped together in the phylogenetic tree (Fig. 2). In one of the cases the transmission of infection was suspected but never established (Fig 2, Z1), while in the other case, the transmission from cat to owner was established by both epidemiological and molecular investigations [11]. In zoonotic case 1 (Z1) the sequences retrieved from both the cat and owner were identical, but differed significantly from all other sequences within our dataset (Cluster B, Fig 2). The sequences retrieved from the cat and owner in zoonotic case 2 (Z2) also differed from all other sequences obtained, but also from each other by one nucleotide in the *gp60* sequence; where all other sequences in our dataset displayed an adenine (A) in position 833, the sequence retrieved from the cat had a guanine (G). This single nucleotide polymorphism resulted in an amino acid change. However, careful inspection of *C. felis* NGS data from this cat (which was the donor of the sample from which the genome sequence used to identify the *gp60* sequence was derived) showed that one of 62 high quality sequence reads had an A in that position. This clearly shows that observed variation and dominant variant within a single *C. felis* isolate shifted when transmitted from one host (cat) to another host (human) (Fig. 3).

### Nomenclature

The subtype family of *C. felis gp60* is designated XIX. Since the gene is so variable and most of the sequences are unique, and no serine tract is present, no specific subtype nomenclature has been established.

## Discussion

The *gp60* gene is currently the most commonly used genetic marker for subtyping of *Cryptosporidium* spp., often using the number of, and variation in, repeats in the serine tract present in most species explored in combination with the adjacent sequence. The serine tract is lacking in the genome of *C. felis* and *C. ubiquitum* [14] as well as in the recently described *C. suis gp60* sequences (GenBank: MH187875 and MH187874). The variability among *Cryptosporidium gp60* sequences indicates that the use of universal primers for *gp60* amplification is inappropriate.

We have identified the *gp60* orthologue in *C. felis* and used a novel, variable region for subtyping (Fig. 1). The *gp60* orthologue in *C. felis* is highly variable and most samples generated fragments that vary greatly in size which renders weak phylogenies and thus gives poorly supported trees under all algorithms tested. However, the marker is very useful to confirm relatedness between samples, as for example in source tracing and suspected zoonotic transmission. This need, to have a distinct subtyping tools for this purpose, was highlighted in the previous report on a possible zoonotic transmission of *C. felis* from a cat to its owner [11]. In this type of case, the diverged nature of the *C. felis gp60* orthologue is useful to trace zoonotic transmissions, since the large variability makes the likelihood of false positive connections low.

Comparing the human and feline isolates stemming from the established zoonotic transmission from a cat to its owner revealed one single nucleotide substitution (A to G). However, for this particular feline isolate, we have NGS data at a 62-fold coverage of the locus of the substitution. Although the majority of reads displayed this feline isolate-specific adenine, one high quality read matched the sequence found in the human-derived isolate. In the DNA sequence generated from Sanger sequencing of some PCR products covering this region there were double peaks in this exact position, further strengthening our observation. Samples with *Cryptosporidium* parasites are more likely to contain mixed populations of oocysts rather than a clonal population, and should be looked on as mixed infections. The variability in an infection may change when the population moves from one host to another which was apparent in the case described herein.

We have detected a large variability among the *gp60* sequences in our dataset. However, there were also some conserved groups. The most striking example is a cluster (Cluster D, Fig. 2) of 17 almost identical sequences, obtained from humans in both Sweden and the UK, many of whom had recently travelled abroad. More strikingly for the cases in this highly conserved group is the overrepresentation of men (16/17 cases), which is unusual as women are generally overrepresented among adults with cryptosporidiosis [19]. Another intriguing observation linked to this cluster is the median age of the group, 37 years-old, which is atypical for a mostly pediatric infection [25]. Furthermore, this group represents the cluster most distinct from the other *gp60* sequences obtained. Altogether our data suggest that these 17 sequences represent a separate, conserved variant. It would be interesting to study other genetic markers among samples from this group.

## Conclusions

We have identified the *gp60* gene in *C. felis* and show how this highly variable marker can be used to supplement epidemiological investigations and aid in source tracking. Due to occasionally occurring double peaks in these sequences it is imperative to have a close look at chromatograms before drawing conclusions.

## Supplementary information

**Additional file 1: Table S1.** Sample origin and GenBank accession number as well as sex and age of each patient.

## Data Availability

All data generated or analysed during this study are included in this published article. All newly generated sequences were deposited in the GenBank database under the accession numbers MH240831-MH240912.

## Abbreviations

*gp60*: 60-kDa glycoprotein
*18S*: 18S ribosomal RNA gene
COWP: *Cryptosporidium* oocyst wall protein
HSP70: heat-shock protein 70
PCR: polymerase chain reaction
Z1: zoonotic case 1
Z2: zoonotic case 2
F1: feline outbreak 1
