# Supplementary material for: Development of a gp60-subtyping method for Cryptosporidium felis
Source: Parasit Vectors. 2020 Jan 23;13:39. doi: 10.1186/s13071-020-3906-9 (PMC6979280; doi:10.1186/s13071-020-3906-9)
Supplement: Supplementary file 1 — Additional file 1: Table S1. Sample origin and GenBank accession number as well as sex and age of each patient. [file 13071_2020_3906_MOESM1_ESM.docx]

**Additional file 1: Table S1.** Sample origin and GenBank accession number as well as sex and age of each patient.

| **Origin** | **Sample** | **Accession number** | **Age** | **Sex** | **Origin of infection** |
| --- | --- | --- | --- | --- | --- |
| Human | UKFEL14 | MH240831 | 72 | M | Sudan |
| Human | UKFEL18 | MH240831 | 41 | M | USA |
| Human | UKFEL23 | MH240831 | 38 | M | UK* |
| Human | UKFEL24 | MH240831 | 29 | M | UK* |
| Human | UKFEL25 | MH240831 | 2 | M | UK* |
| Human | UKFEL26 | MH240831 | 2 | M | Pakistan |
| Human | UKFEL34 | MH240831 | 55 | M | UK* |
| Human | UKFEL35 | MH240831 | 33 | M | UK* |
| Human | UKFEL37 | MH240831 | 37 | M | UK* |
| Human | UKFEL38 | MH240831 | 35 | M | UK* |
| Human | Swec053 | MH240831 | 37 | M | India |
| Human | UKFEL42 | MH240831 | 21 | M | India |
| Human | UKFEL75 | MH240831 | 65 | M | UK* |
| Human | UKFEL77 | MH240831 | 44 | M | UK* |
| Human | UKFEL80 | MH240831 | 19 | F | India |
| Human | UKFEL82 | MH240831 | 40 | M | UK* |
| Human | UKFEL88 | MH240831 | 52 | M | UK* |
| Human | UKFEL85 | MH240832 | 28 | F | Dominican republic |
| Human | UKFEL4 | MH240833 | 50 | M | UK* |
| Human | UKFEL70 | MH240834 | 44 | F | UK* |
| Human | UKFEL47 | MH240835 | 21 | F | UK* |
| Human | UKFEL27 | MH240836 | 18 | F | UK* |
| Human | UKFEL33 | MH240837 | 7 | M | UK* |
| Human | UKFEL64 | MH240838 | 54 | F | UK* |
| Human | UKFEL22 | MH240839 | 57 | M | UK* |
| Human | UKFEL79 | MH240840 | 41 | M | UK* |
| Human | UKFEL66 | MH240841 | 21 | F | UK* |
| Human | UKFEL30 | MH240842 | 23 | F | UK* |
| Human | UKFEL59 | MH240843 | 6 | M | UK* |
| Human | UKFEL67 | MH240844 | 9 | F | UK* |
| Human | UKFEL69 | MH240845 | 1 | M | UK* |
| Human | UKFEL73 | MH240846 | 40 | F | UK* |
| Human | UKFEL61 | MH240847 | 23 | F | UK* |
| Cat | Cat730 | MH240848 |  |  | Denmark |
| Cat | Cat744 | MH240849 |  |  | Denmark |
| Cat | Cat4D | MH240850 |  |  | Denmark |
| Cat | Cat736 | MH240851 |  |  | Denmark |
| Human | Swec529 | MH240852 | 19 | F | Indonesia |
| Human | UKFEL3 | MH240853 | 35 | F | UK* |
| Cat | Cat6A | MH240854 |  |  | Denmark |
| Cat | Cat621 | MH240855 |  |  | Denmark |
| Human | Swec459 | MH240856 | 46 | M | Sweden |
| Cat | Cat197 | MH240857 |  |  | Denmark |
| Human | UKFEL44 | MH240858 | 22 | F | UK* |
| Human | UKFEL10 | MH240859 | 6 | M | UK* |
| Cat | SWEFEL1 | MH240860 |  |  | Sweden |
| Human | UKFEL12 | MH240861 | 34 | F | Spain |
| Human | UKFEL36 | MH240862 | 9 | F | UK* |
| Human | UKFEL83 | MH240863 | 4 | M | UK* |
| Human | UKFEL57 | MH240864 | 25 | M | UK* |
| Human | UKFEL63 | MH240865 | 40 | F | UK* |
| Human | UKFEL58 | MH240866 | 66 | F | UK* |
| Human | UKFEL71 | MH240867 | 6 | F | UK* |
| Human | UKFEL21 | MH240868 | 8 | F | UK* |
| Human | UKFEL15 | MH240869 | 10 | M | UK* |
| Human | UKFEL49 | MH240870 | 16 | M | UK* |
| Human | UKFEL41 | MH240871 | 20 | F | UK* |
| Human | UKFEL62 | MH240872 | 24 | M | Peru |
| Cat | Cat12D | MH240873 |  |  | Denmark (F1) |
| Cat | Cat 15D | MH240873 |  |  | Denmark (F1) |
| Cat | Cat17D | [MH240873](https://www.ncbi.nlm.nih.gov/nucleotide/MH240873.1?report=genbank&log$=nuclalign&blast_rank=1&RID=19G24JEW014) |  |  | Denmark (F1) |
| Human | Swec325 | MH240874 | 37 | F | Sweden |
| Cat | Cat11D | MH240875 |  |  | Denmark (F1) |
| Human | Swec156 | MH240876 | 37 | F | Sweden |
| Cat | Swecat502 | [MH240877](https://www.ncbi.nlm.nih.gov/nucleotide/MH240877.1?report=genbank&log$=nuclalign&blast_rank=1&RID=189MP1EU01R) |  |  | Sweden |
| Cat | Swecat501 | MH240877 |  |  | Sweden |
| Human | UKFEL16 | MH240878 | 1 | F | Croatia |
| Cat | Swecat212 | MH240879 |  |  | Sweden |
| Cat | Swecat180 | MH240880 |  |  | Sweden |
| Cat | Cat287 | MH240881 |  |  | Denmark |
| Human | UKFEL39 | MH240882 | 25 | M | Croatia |
| Human | SC142 | MH240883 | 37 | F | Sweden (Z2) |
| Cat | Swecat113 | MH240884 |  |  | Sweden (Z2) |
| Human | UKFEL60 | MH240885 | 36 | F | UK* |
| Human | Swec206 | MH240886 | 30 | F | Sweden |
| Cat | Swecat977 | MH240887 |  |  | Sweden |
| Human | UKFEL52 | MH240888 | 4 | F | UK* |
| Human | UKFEL53 | MH240889 | 22 | F | UK* |
| Human | UKFEL68 | MH240890 | 1 | F | UK* |
| Human | UKFEL78 | MH240891 | 7 | M | UK* |
| Human | UKFEL51 | MH240892 | 27 | M | UK* |
| Human | UKFEL43 | MH240893 | 16 | F | UK* |
| Human | UKFEL55 | MH240894 | 40 | M | UK* |
| Human | UKFEL72 | MH240895 | 75 | M | UK* |
| Human | UKFEL56 | MH240896 | 1 | M | UK* |
| Human | UKFEL8 | MH240897 | 36 | M | UK* |
| Human | UKFEL65 | MH240898 | 20 | F | UK* |
| Human | UKFEL46 | MH240899 | 33 | M | UK* |
| Human | UKFEL31 | MH240900 | 1 | F | UK* |
| Human | Swec637 | MH240901 | 40 | F | Sweden (Z1) |
| Cat | Swecat114 | MH240901 |  |  | Sweden (Z1) |
| Cat | Swecat503 | MH240902 |  |  | Sweden |
| Cat | Swecat153 | MH240903 |  |  | Sweden |
| Human | UKFEL28 | MH240904 | 5 | M | UK* |
| Human | UKFEL74 | MH240905 | 26 | F | UK* |
| Human | UKFEL17 | MH240906 | 2 | M | UK* |
| Human | UKFEL76 | MH240907 | 27 | M | UK* |
| Human | UKFEL87 | MH240908 | 2 | F | UK* |
| Human | UKFEL45 | MH240909 | 24 | M | UK* |
| Human | UKFEL2 | MH240910 | 6 | M | UK* |
| Human | UKFEL81 | MH240911 | 4 | M | UK* |
| Human | UKFEL13 | MH240912 | 30 | M | UK* |

*** samples with unknown travel history

*Abbreviation*: M, male; F, female; Z2, zoonotic case 2; Z1, zoonotic case 1; F1, feline outbreak 1
